# Intravenous glutamine decreases lung and distal organ injury in an experimental model of abdominal sepsis

**DOI:** 10.1186/cc7888

**Published:** 2009-05-19

**Authors:** Gisele P Oliveira, Mariana BG Oliveira, Raquel S Santos, Letícia D Lima, Cristina M Dias, Alexandre M AB' Saber, Walcy R Teodoro, Vera L Capelozzi, Rachel N Gomes, Patricia T Bozza, Paolo Pelosi, Patricia RM Rocco

**Affiliations:** 1 Laboratory of Pulmonary Investigation, Carlos Chagas Filho Institute of Biophysics, Federal University of Rio de Janeiro, Av. Carlos Chagas Filho, s/n, Rio de Janeiro, 21949-902, Brazil; 2 Department of Pathology, Faculty of Medicine, University of São Paulo, Dr. Arnaldo Street, 455, Sao Paulo, 01246-903, Brazil; 3 Laboratory of Immunopharmacology, Oswaldo Cruz Institute, FIOCRUZ, Avenida Brasil 4365, Rio de Janeiro, 21045-900, Brazil; 4 Department of Ambient, Health and Safety, University of Insubria, c/o Villa Toeplitz Via G.B. Vico, 46 21100 Varese, Italy

## Abstract

**Introduction:**

The protective effect of glutamine, as a pharmacological agent against lung injury, has been reported in experimental sepsis; however, its efficacy at improving oxygenation and lung mechanics, attenuating diaphragm and distal organ injury has to be better elucidated. In the present study, we tested the hypothesis that a single early intravenous dose of glutamine was associated not only with the improvement of lung morpho-function, but also the reduction of the inflammatory process and epithelial cell apoptosis in kidney, liver, and intestine villi.

**Methods:**

Seventy-two Wistar rats were randomly assigned into four groups. Sepsis was induced by cecal ligation and puncture surgery (CLP), while a sham operated group was used as control (C). One hour after surgery, C and CLP groups were further randomized into subgroups receiving intravenous saline (1 ml, SAL) or glutamine (0.75 g/kg, Gln). At 48 hours, animals were anesthetized, and the following parameters were measured: arterial oxygenation, pulmonary mechanics, and diaphragm, lung, kidney, liver, and small intestine villi histology. At 18 and 48 hours, Cytokine-Induced Neutrophil Chemoattractant (CINC)-1, interleukin (IL)-6 and 10 were quantified in bronchoalveolar and peritoneal lavage fluids (BALF and PLF, respectively).

**Results:**

CLP induced: a) deterioration of lung mechanics and gas exchange; b) ultrastructural changes of lung parenchyma and diaphragm; and c) lung and distal organ epithelial cell apoptosis. Glutamine improved survival rate, oxygenation and lung mechanics, minimized pulmonary and diaphragmatic changes, attenuating lung and distal organ epithelial cell apoptosis. Glutamine increased IL-10 in peritoneal lavage fluid at 18 hours and bronchoalveolar lavage fluid at 48 hours, but decreased CINC-1 and IL-6 in BALF and PLF only at 18 hours.

**Conclusions:**

In an experimental model of abdominal sepsis, a single intravenous dose of glutamine administered after sepsis induction may modulate the inflammatory process reducing not only the risk of lung injury, but also distal organ impairment. These results suggest that intravenous glutamine may be a potentially beneficial therapy for abdominal sepsis.

## Introduction

Sepsis is the most important risk factor for acute lung injury (ALI)/acute respiratory distress syndrome (ARDS) [1] and can trigger long-term consequences. Overwhelming inflammatory and immune responses are fundamental features of sepsis and are known to play a crucial role in the pathogenesis of hypotension, tissue damage, multiple organ dysfunction syndrome, and death.

Levels of glutamine (Gln), a non-essential amino acid, have been demonstrated to decrease during critical illness, mainly in sepsis [2-4]. Additionally, lower levels of Gln have also been associated with immune dysfunction [2,5] and higher mortality rate [6,7]. In this line, many clinical [8-10] and experimental [11-17] studies have suggested that intravenous (iv) Gln may prevent the occurrence of lung injury, tissue metabolic dysfunction, improving survival after sepsis. The mechanism by which Gln attenuates pro-inflammatory cytokines and improves patient outcome has been extensively investigated [17-19]. Gln can enhance stress-inducible heat shock protein (HSP) expression, such as HSP 70 [12,13,17,18], and suppress nuclear factor-κB (NF-κB) signal transduction activity [11,19], decreasing neutrophil infiltration and production of cytokines [11,19,20]. However, no previous studies have evaluated the impact of iv Gln at improving oxygenation and lung mechanics, attenuating diaphragm and distal organ injury in sepsis [20].

In the present study, we tested the hypothesis that a single early iv dose of Gln was associated not only with the improvement of lung morpho-function, but also the reduction of the inflammatory process and epithelial cell apoptosis in kidney, liver, and intestine villi in an experimental model of abdominal sepsis. For this purpose, we evaluated the effects of Gln on partial pressure of arterial oxygen (PaO_2_), lung mechanics, and histology (light, electron and confocal microscopy, and apoptosis), electron microscopy of diaphragm, and histology and epithelial cell apoptosis in kidney, liver, and small intestine villi. Additionally, the balance of pro- and anti-inflammatory cytokines in bronchoalveolar lavage fluids (BALF) and peritoneal lavage fluids (PLF) were analysed.

## Materials and methods

### Animal preparation and experimental protocol

This study was approved by the Ethics Committee of the Carlos Chagas Filho Institute of Biophysics, Health Sciences Centre, and Federal University of Brazil. All animals received humane care in compliance with the *Principles of Laboratory Animal Care *formulated by the National Society for Medical Research and the *Guide for the Care and Use of Laboratory Animals *prepared by the US National Academy of Sciences.

A total of 72 adult male Wistar rats (weighing 230 to 250 g) were randomly assigned into two main groups: cecal ligation and puncture-induced sepsis (CLP, n = 36) [20]; and control (C, n = 36), a sham-operated group. One hour after surgery, C and CLP groups were further randomized into subgroups receiving iv saline (1 ml, SAL, n = 18 per group) or Gln (0.75 g/kg body weight, 1 ml iv, Gln, n = 18 per group) through a lateral tail vein. Gln was administered as an alanyl-Gln dipeptide (Dipeptiven 20%^®^, Fresenius Kabi Brazil, LTDA Campinas, São Paulo, Brazil). Pulmonary mechanics and the histology of lung, diaphragm, liver, kidney, and small intestine villi were studied in eight animals per group at 48 hours and the amount of cytokines in PLF and BALF were analysed in five animals per group at 18 and 48 hours.

Animals were fasted for 16 hours before any surgical procedure to create similar bowel contents. Rats were anesthetized with sevoflurane, a midline laparotomy (2 cm incision) was performed, the cecum was carefully isolated to avoid damage to blood vessels, and a 3.0 cotton ligature was placed around the cecum just below the ileocecal valve to avoid bowel obstruction. In the CLP group, the cecum was punctured twice with an 18 gauge needle [21]. In sham-operated group, an abdominal incision was made with no cecal ligation and perforation. Both layers of abdominal cavity were closed with 3.0 silk sutures, followed by fluid resuscitation (20 ml/kg body weight of sterile saline, subcutaneously) [21].

Forty-eight hours after surgery, rats were sedated (diazepam 5 mg, intraperitoneally (ip)), anaesthetised (thiopental sodium 20 mg/kg, ip), tracheotomised, paralysed (pancuronium bromide 1 mg/kg, iv), and ventilated with a constant flow ventilator (Samay VR15; Universidad de la Republica, Montevideo, Uruguay) with the following parameters: tidal volume (V_T_) = 6 mL/kg, constant airflow = 7 mL/sec, frequency = 100 breaths/min, inspiratory to expiratory ratio = 1:2, fraction of inspired oxygen (FiO_2_) = 0.21, and positive end-expiratory pressure (PEEP) = 5 cmH_2_O. A polyethylene catheter (PE-10) was introduced into the femoral artery for blood sampling. Blood (300 μL) was drawn into a heparinised syringe for PaO_2 _(i-STAT, Abbott Laboratories, North Chicago, IL, USA). After a 15-minute ventilation period, PaO_2 _was measured and lung mechanics computed. Lungs, liver, kidneys, small intestine villi, and diaphragm were then prepared for histology.

### Respiratory mechanics

A pneumotachograph was connected to the tracheal cannula for the measurements of airflow (V'). The pressure gradient across the pneumotachograph was determined by means of a differential pressure transducer (SCIREQ, SC-24, Montreal, Quebec, Canada). V_T _was obtained by integration of the V' signal. The flow resistance of the equipment (Req), tracheal cannula included, was constant up to flow rates of 26 mL/s, and amounted to 0.12 cmH_2_O/mL/s. Equipment resistive pressure (Req/V') was subtracted from pulmonary resistive pressure so that the results represent intrinsic values. Tracheal pressure was also measured with a differential pressure transducer (SCIREQ, SC-24, Montreal, Quebec, Canada). Changes in oesophageal pressure, which reflect chest wall pressure, were measured with a 30 cm long water-filled catheter (PE205) with side holes at the tip connected to a SCIREQ differential pressure transducer (SCIREQ, SC-24, Montreal, Quebec, Canada). Transpulmonary pressures were calculated by the difference between tracheal and oesophageal pressures [22]. All signals were filtered (100 Hz), amplified in a four-channel conditioner (SCIREQ, SC-24, Montreal, Quebec, Canada), sampled at 200 Hz with a 12-bit analogue-to-digital converter (DT2801A, Data Translation, Marlboro, MA, USA), and stored on a microcomputer. All data were collected using LABDAT software (RHT-InfoData, Montreal, Quebec, Canada).

Lung resistive pressure (ΔP1), viscoelastic/inhomogeneous (ΔP2) pressure, and static elastance (Est) were computed by the end-inflation occlusion method [23]. Briefly, after end-inspiratory occlusion there is an initial fast drop in pressure from the preocclusion value (peak inspiratory pressure) down to an inflection point (ΔP1), followed by slow pressure decay (ΔP2), until a plateau (Pplat, L) is reached. This plateau corresponds to the lung elastic recoil pressure. ΔP1 selectively reflects airway resistance and ΔP2 reflects lung viscoelastic properties together with a small contribution of time-constant inhomogeneities. Est was calculated by dividing Pplat, L by the V_T_. Pulmonary mechanics measurements were performed 10 times in each animal, and analyzed using ANADAT data analysis software (RHT-InfoData Inc., Montreal, Quebec, Canada).

### Light microscopy

A laparotomy was performed immediately after the determination of lung mechanics (END) and heparin (1000 IU) was intravenously injected in the vena cava. The trachea was clamped at 5 cmH_2_O PEEP, and the abdominal aorta and vena cava were sectioned, yielding a massive haemorrhage that quickly killed the animals. Then, the lungs were removed *en bloc *at the same PEEP in all groups to avoid distortion of lung morphometry. The right lung was immersed in 3% buffered formaldehyde. Liver, kidneys, and small intestine were also removed, immersed in 3% buffered formaldehyde, and paraffin embedded. Four-μm-thick slices were cut and stained with H&E.

Lung morphometric analysis was performed with an integrating eyepiece with a coherent system consisting of a grid with 100 points and 50 lines (known length) coupled to a conventional light microscope (Olympus BX51, Olympus Latin America-Inc., São Paulo, Brazil). The volume fraction of the lung occupied by hyperinflated structures (alveolar ducts, alveolar sacs, or alveoli wider than 120 μm) or collapsed alveoli or normal pulmonary areas were determined by the point-counting technique [24] at a magnification of 200× across 10 random, non-coincident microscopic fields [22].

### Transmission electron microscopy

Three slices of 2 × 2 × 2 mm were cut from three different segments of the left lung and diaphragm. They were then fixed for electron microscopy analysis. For each electron microscopy image (20 per animal) an injury score was determined. The following parameters were analyzed concerning lung parenchyma: type II epithelial cell lesion; hyaline membrane; and endothelial cell damage [22]. The following data were obtained from the electron microscopy of diaphragm muscle: oedema of Z-disc and mitochondrial injury. The pathologic findings were graded according to a five-point semi-quantitative severity-based scoring system as follows: 0 = normal lung parenchyma or diaphragm, 1 = changes in 1 to 25%, 2 = changes in 26 to 50%, 3 = changes in 51 to 75%, and 4 = changes in 76 to 100% of examined tissue.

### Confocal microscopy

Anti-Thyroid Transcription Factor 1 (TTF1) and anti-CD34 fluorescence immunohistochemistry were respectively used to analyze epithelial and endothelial components of the alveolar barrier using confocal microscopy. Cells were incubated with anti-TTF1 (monoclonal antibody, Santa Cruz Biotechnology, Santa Cruz, CA, USA, 1:25) and anti-CD34 (monoclonal antibody, Novocastra Laboratories Ltd., Newcastle upon Tyne, UK, 1:400), followed by double staining with fluorescein and rhodamine (rhodamine-conjugated goat anti-mouse IgG-R, dilution 1:40, Santa Cruz Biotechnology, Santa Cruz, CA, USA). Images were obtained using a Zeiss LSM-410 laser-scanning confocal microscope (Carl Zeiss Canada Ltd, Toronto, ON, Canada) [25].

### Apoptosis assay of lung and distal organs

Apoptotic cells of lung, kidney, liver, and small intestine villi were quantified using the Terminal deoxynucleotidyl Transferase Biotin-dUTP Nick End Labelling (TUNEL) assay [26] and immunohistochemical staining for Fas and FasL protein [27].

To detect DNA fragmentation in cell nuclei, TUNEL reaction was applied to the paraffin sections by using *In Situ *Cell Death Detection Kit, Fluorescin (Boehringer, Mannheim, Germany). Formalin fixed and paraffin-embedded lung tissue sections were deparaffinized and antigen retrieval was carried out by incubating tissue slides with protein kinase K (Roche Applied Science, Indianapolis, IN, USA) for 20 minutes at 15 μg/ml. TUNEL reaction mixture was applied for one hour at 37°C. For negative controls the transferase enzyme was omitted. The nuclei without DNA fragmentation stained blue as a result of counterstaining with hematoxylin. Positive controls consisted of rat prostatic gland after castration.

The cellular localization of Fas and FasL proteins was studied by the streptavidin-biotin immunoperoxidase method using a polyclonal rabbit anti-FasL antibody (Chemicon/Millipore, Billerica, MA, USA). Immunoreactivity was detected with 3,3'-diaminobenzidine tetrachloride. Specificity controls consisted of omission of primary antibody and/or preabsorption with blocking peptide, which abolished all immunoreactivity.

Three sections from each specimen were initially examined under light microscopy at low magnification (× 100), allowing the evaluation of surface area occupied by apoptotic cells. Then, 10 fields per section were randomly examined at a higher magnification (× 400). A five-point semi-quantitative severity-based scoring system was used and graded as: 0 = no apoptotic cells; 1 = 1 to 25%; 2 = 26 to 50%; 3 = 51 to75%; 4 = 76 to 100% of apoptotic cells in the examined tissue.

Two investigators, unaware of the origin of the material, examined the samples microscopically. The slides were coded and examined only at the end of all measurements.

### Peritoneal and bronchoalveolar lavage fluids

Another 20 rats (n = 5 per group) were submitted to the same protocol previously described to obtain aliquots of PLF and BALF at 18 and 48 hours after surgery. Amounts of Cytokine-Induced Neutrophil Chemoattractant (CINC-1), and IL-6 and 10 were quantified by ELISA according to manufacturer's protocol (Duo Set, R&D Systems, Minneapolis, MN, USA).

### Statistical analysis

SigmaStat 3.1 statistical software package (Jandel Corporation, San Raphael, CA, USA) was used. Differences among the groups were assessed by a two-way analysis of variance (ANOVA) followed by Tukey's test when required. Nonparametric data were analyzed using a two-way ANOVA on ranks followed by Dunn's *post hoc *test. The parametric data were expressed as mean ± standard deviation, while the non-parametric data were expressed as median (interquartile range). A *P *< 0.05 was considered significant.

## Results

In pilot studies we determined that this CLP model of sepsis resulted in an approximate 60% survival rate at 48 hours. A single dose of Gln (0.75 g/kg body weight iv), one hour after the CLP surgery, significantly increased (*P *< 0.05) survival (100%) at 48 hours (CLP-Gln). No deaths occurred in the C group.

CLP-SAL showed lower PaO_2 _(55 ± 6 mmHg) than C-SAL (91 ± 8 mmHg). PaO_2 _was significantly (*P *< 0.05) higher in CLP-Gln than CLP-SAL (86 ± 6 mmHg *vs *55 ± 6 mmHg), and a similar result was seen in C-SAL and C-Gln (from 91 ± 8 mmHg to 87 ± 4 mmHg).

**Figure 1:**
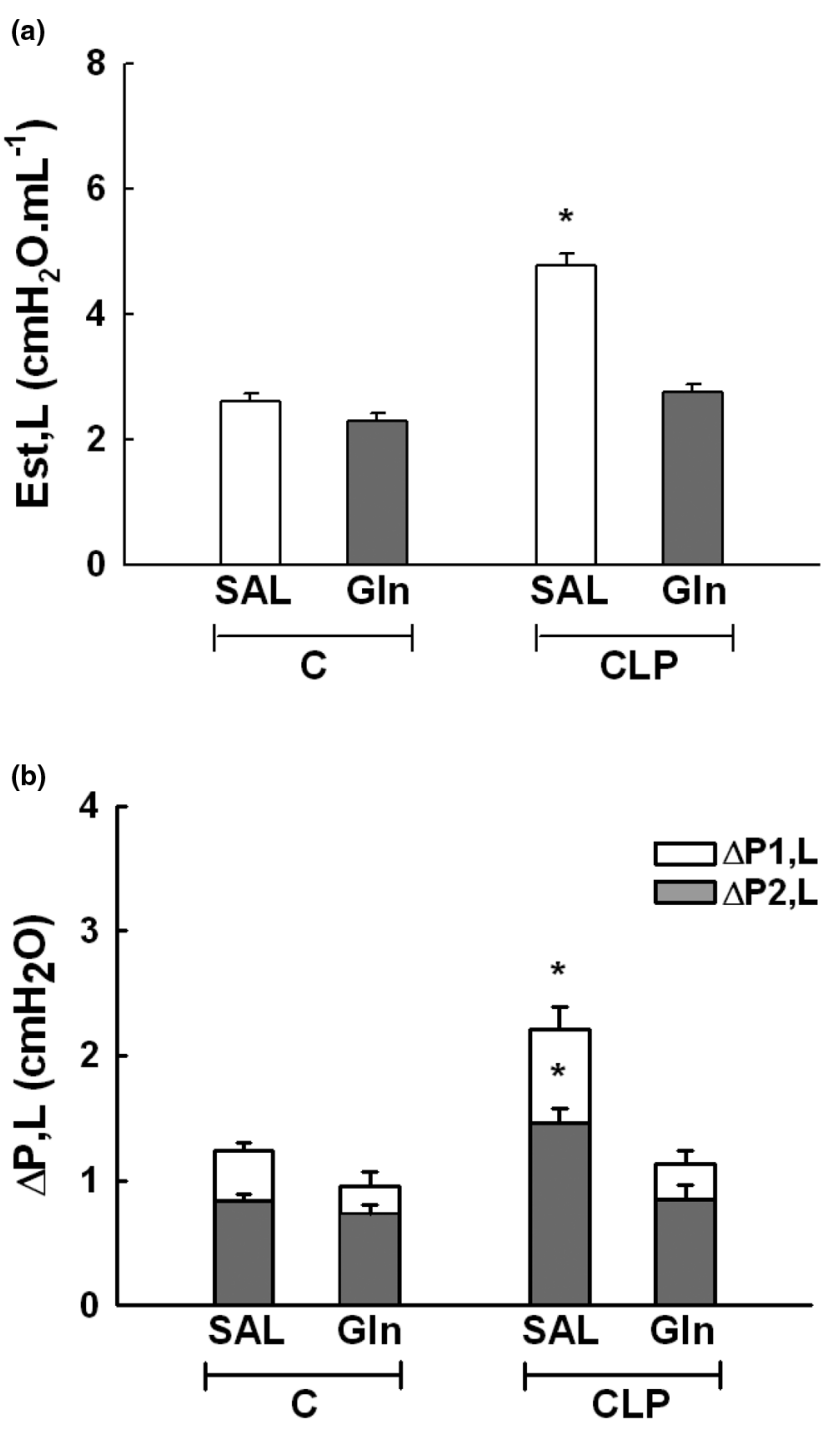
Means ± standard deviation of eight animals in each group (10 determinations per animal).  **(a) **Lung static elastance (Est, L) measures are shown. **(b) **Stacked bars chart plot data in which white bars represent the lung viscous pressure (ΔP1, L) and gray bars are the viscoelastic/inhomogeneous (ΔP2, L) pressure dissipations. The whole column represents the total pressure (ΔPtot, L) variation in each group. Sepsis was induced by cecal ligation and puncture surgery (CLP). A sham-operated group was used as control group (C) for animals undergoing CLP. One hour after surgery, C and CLP groups were treated with saline (SAL) or glutamine (Gln). *Significantly different from C-SAL group (*P *< 0.05).

There were no significant differences in flow, V_T _as well as chest wall mechanical data among groups. Lung Est (+ 71%), ΔP1 (+ 28%), and ΔP2 (+ 64%) were increased in CLP-SAL as compared with C-SAL (Figures 1a and 1b). CLP-Gln showed lung mechanical data similar to C-Gln (Figures 1a and 1b).

**Figure 2:**
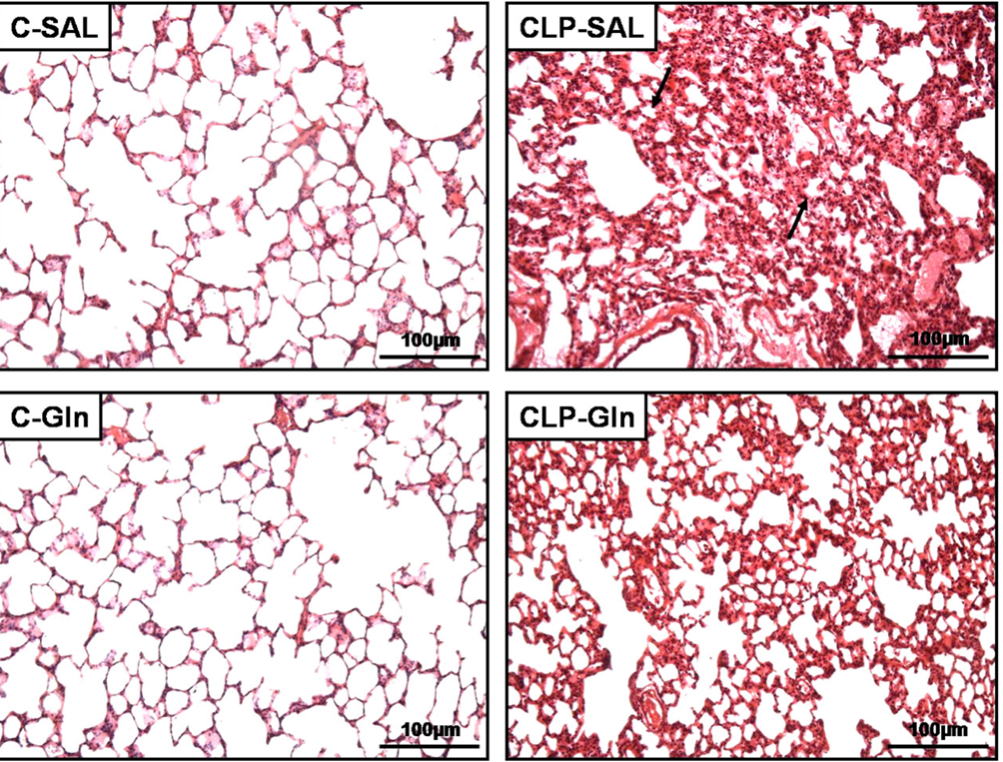
Representative photomicrographs of lung parenchyma in C-SAL, C-Gln, CLP-SAL and CLP-Gln.  In CLP group, animals were submitted to cecal ligation and puncture technique. A sham-operated group was used as control (C) for animals undergoing CLP. One hour after surgery, C and CLP groups were treated with saline (SAL) or glutamine (Gln). Note the areas of alveolar collapse (arrows). Photomicrographs were taken at an original magnification of × 200 from slides stained by haematoxylin & eosin.

**Figure 3:**
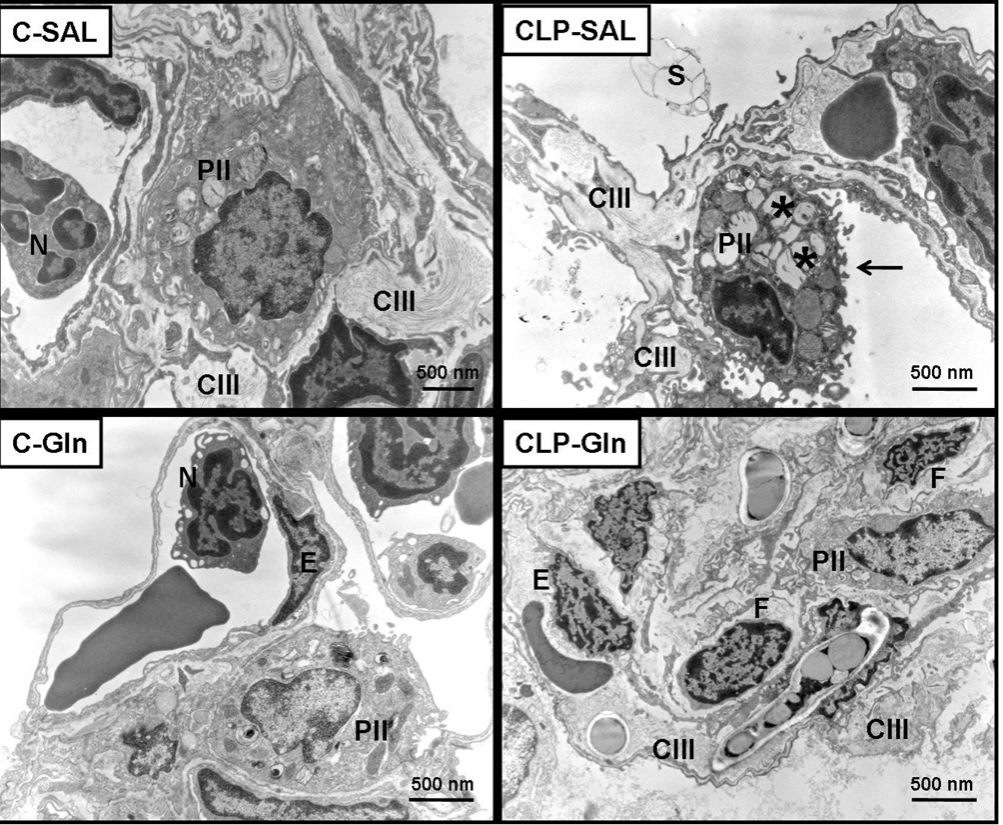
Electron microscopy of lung parenchyma.  Type II pneumocyte was well preserved with integrity of lamellar bodies and typical microvilli projecting from its surface in C-SAL, C-Gln and CLP-Gln groups. Neutrophils (N); type III collagen fibres (CIII); type II pneumocytes (PII); surfactant molecule (S); endothelial cell (E); fibroblast (F). *Degeneration of lamellar bodies. Note the damage in microvilli of type II pneumocyte in CLP-SAL group (arrow). Photomicrographs are representative of data obtained from lung section derived from five animals. C = control; CLP = cecal ligation and puncture; Gln = glutamine; SAL = saline.

**Figure 4:**
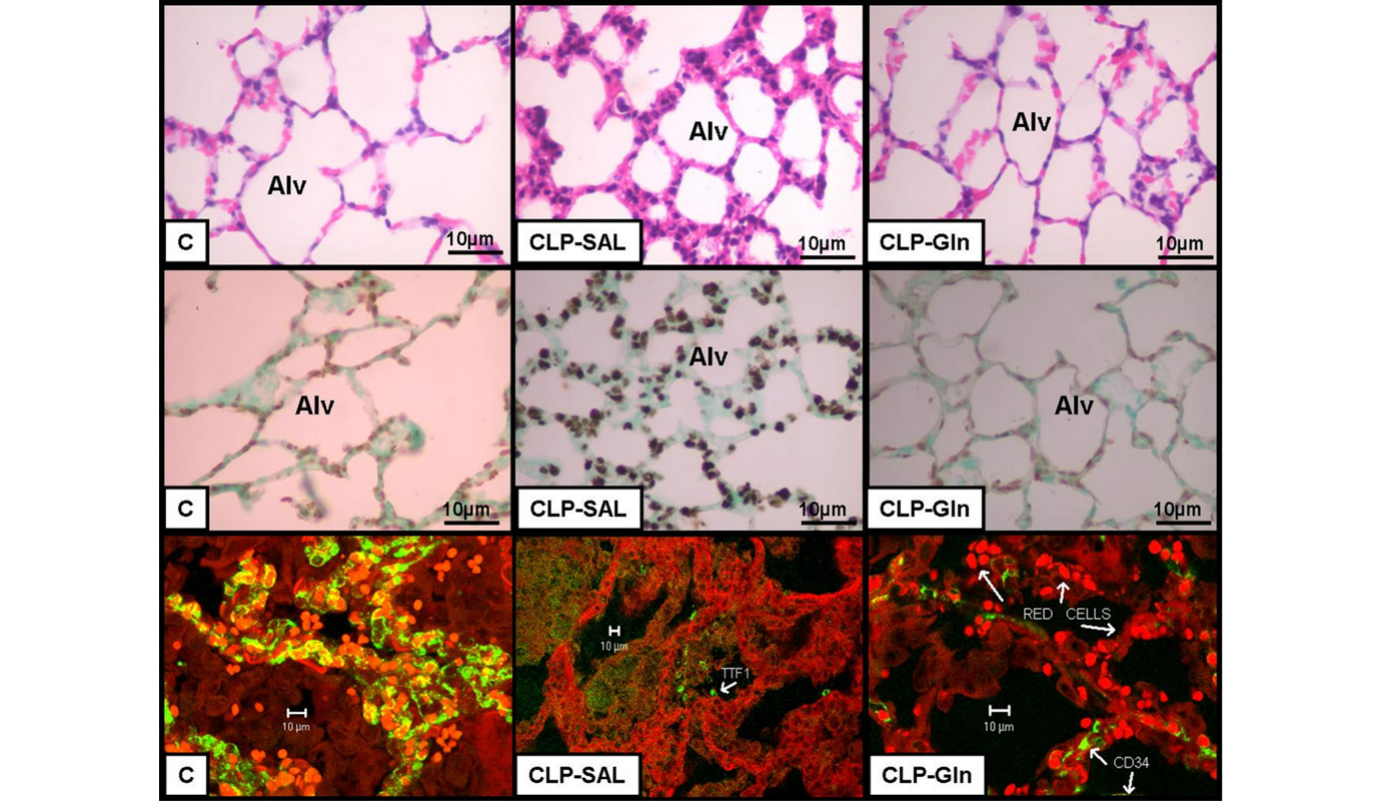
Representative photomicrographs of lung parenchyma.  Samples were stained with **(top) **haematoxylin & eosin, **(middle) **TUNEL, and **(bottom) **double immunofluorescence for TTF1 (Thyroid Transcription Factor 1, alveolar epithelium) and CD34 (endothelium). Control lung (C) shows thin alveolar septa (Alv) with sparse apoptotic cells and normal histoarchitecture after tridimensional reconstruction of confocal microscopy. Positive staining is indicated by black-brown and the contrast background staining is green. CLP-SAL lung presented thickened alveolar septa with inflammatory cells, numerous brownish alveolar apoptotic cells and distortion of the architecture after tridimensional reconstruction at confocal microscopy. Note the regeneration and decreased apoptosis of alveolar epithelial cells after glutamine treatment and restoration of the acinar architecture by tridimensional reconstruction at confocal microscopy (CLP-Gln). Photomicrographs are representative of data obtained from lung sections derived from five animals. CLP = cecal ligation and puncture; Gln = glutamine; SAL = saline.

**Figure 5:**
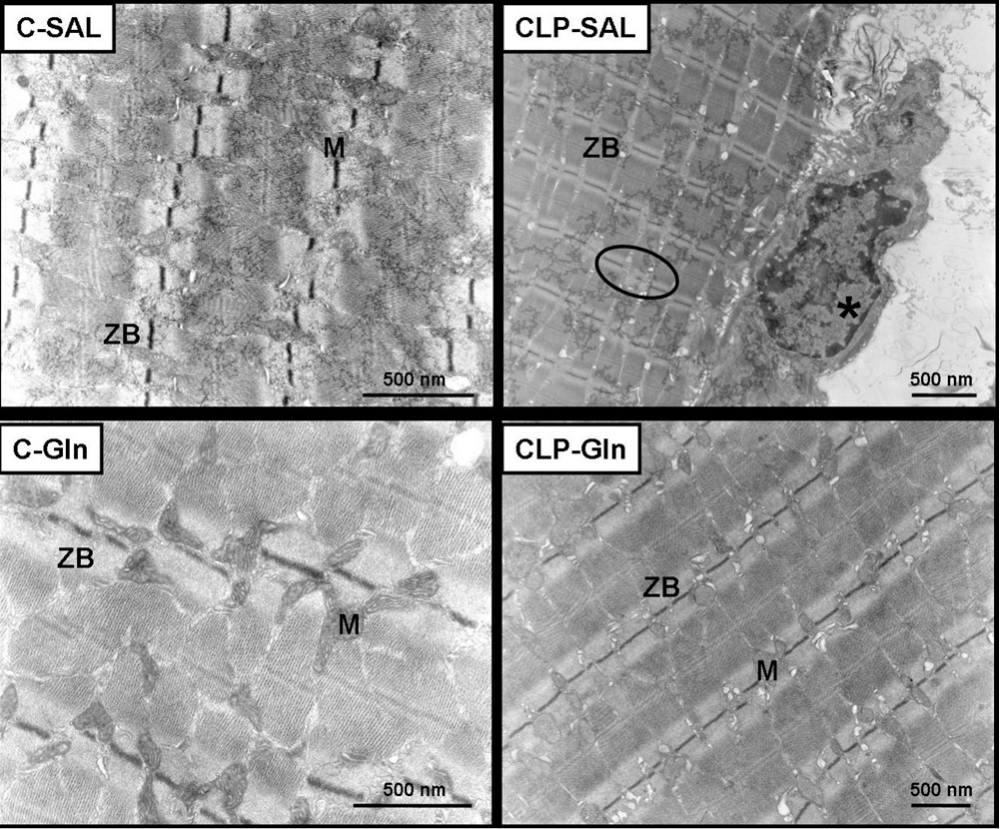
Photomicrographs of electron microscopy of diaphragm.  In C-SAL, C-Gln, and CLP-Gln groups the mitochondria (M) and Z bands (ZB) are well preserved. Asterisk indicates apoptosis in nucleus of muscle. Note the presence of disorganized Z bands (circle) and oedema between muscle fibres in CLP-SAL group. Photomicrographs are representative of data obtained from diaphragm section derived from five animals. C = control; CLP = cecal ligation and puncture; Gln = glutamine; SAL = saline.

In CLP-SAL, lung histology presented neutrophil infiltration, alveolar collapse, interstitial oedema (Table 1 and Figure 2), distortion of lung parenchymal structure, degeneration of lamellar bodies, damage in microvilli, and apoptosis in type II pneumocytes (Figure 3). Note in CLP-Gln regeneration and restoration of the acinar architecture (Figure 3 and Table 2) with tridimensional reconstruction at confocal microscopy (Figure 4). Electronic microscopy of the diaphragm showed oedema between muscle fibres, mitochondrial injury, and apoptosis in muscle cells (Figure 5 and Table 2), while Gln attenuated these morphological changes (Figure 5).

**Table 1:** Lung morphometric parameters

Groups	Normal area (%)	Alveolar collapse (%)	MN (%)	PMN (%)
C-SAL	92.7 ± 0.7	7.3 ± 0.7	36.2 ± 0.4	7.8 ± 0.3
C-Gln	88.3 ± 1.8	11.7 ± 1.8	36.2 ± 0.4	7.7 ± 0.3
CLP-SAL	27.6 ± 3.0*	72.4 ± 3.0*	17.5 ± 1.5*	42.2 ± 1.3*
CLP-Gln	80.7 ± 2.6* #	19.3 ± 2.6* #	31.9 ± 0.6* #	12.9 ± 0.7* #

Values are means (± standard deviation) of eight animals in each group. All values were computed in 10 random, non-coincident fields per rat. The volume fraction of the lung occupied by normal pulmonary areas or collapsed alveoli. Fractional areas of polymorphonuclear cells (PMN) and mononuclear cells (MN). Sepsis was induced by cecal ligation and puncture surgery (CLP). A sham-operated group was used as control (C) for animals undergoing CLP. One hour after surgery, C and CLP groups were treated with saline (SAL) or glutamine (Gln). *Significantly different from C-SAL group (*P *< 0.05). #Significantly different from CLP-SAL group (*P *< 0.05).

**Table 2:** Semi-quantitative analysis of lung and diaphragm electron microscopy

Groups	Type II epithelial cell lesion	Hyaline membrane	Endothelial cell damage	Oedema of Z-disc	Diaphragm mitochondrial injury
C-SAL	0 (0 to 1)	0 (0 to 0)	0 (0 to 0)	0 (0 to 0)	0 (0 to 0)
C-Gln	0 (0 to 0.25)	0 (0 to 0)	0 (0 to 0)	0 (0 to 0)	0 (0 to 0)
CLP-SAL	3 (2 to 3)*	2 (2 to 3)*	4 (3 to 4)*	3 (2 to 4)*	3 (2 to 3)*
CLP-Gln	1 (1 to 2)* #	0 (0 to 1)	1 (1 to 2)* #	0 (0 to 1)	1 (0 to 1)* #

Values are median (25^th ^to 75^th ^percentile) of five rats in each group. The pathologic findings were graded according to a five-point semi-quantitative severity-based scoring system: 0 = normal lung parenchyma or diaphragm, 1 = changes in 1 to 25%, 2 = changes in 26 to 50%, 3 = changes in 51 to 75%, and 4 = changes in 76 to 100% of the examined tissue. Sepsis was induced by cecal ligation and puncture surgery (CLP). A sham-operated group was used as control (C) for animals undergoing CLP. One hour after surgery, C and CLP groups were treated with saline (SAL) or glutamine (Gln). *Significantly different from C group (*P *< 0.05). #Significantly different from CLP-SAL group (*P *< 0.05).

**Figure 6:**
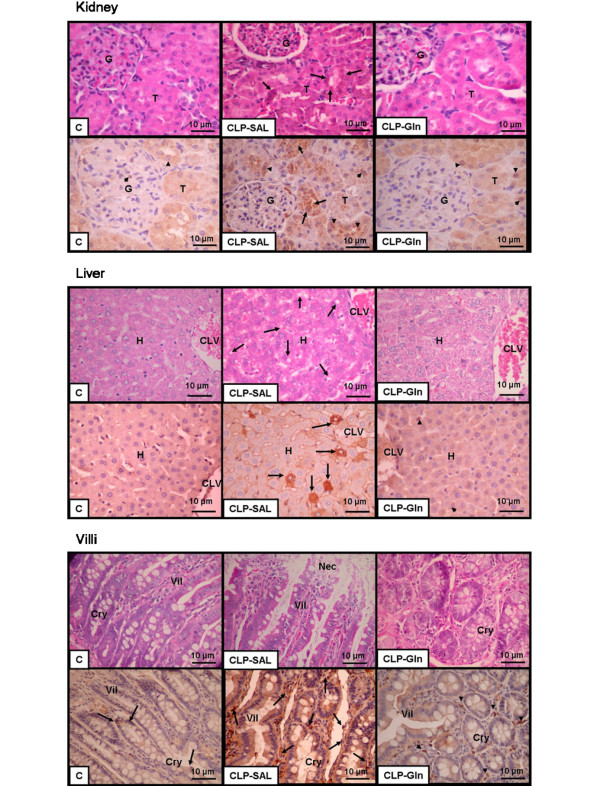
Representative photomicrographs of kidney, liver and small intestine villi stained with (upper panels) haematoxylin & eosin and (lower panels) immunohistochemical staining for FasL.  **(Kidney)**. Control (C) group shows glomeruli (G) and renal tubules (T) with preserved architecture and sparse apoptotic renal cells (arrowheads). Cecal ligation and puncture (CLP) group presents disarrangement of renal tubules with degenerative cytoplasmic changes (arrows) and numerous apoptotic cells. Note in CLP group treated with glutamine (Gln) that the histoarchitecture of the renal tubules is restored with a decrease in apoptotic cells (arrowheads). **(Liver) **C group shows hepatocytes (H) adjacent to centro-lobular vein (CLV) with preserved architecture and few apoptotic cells. In CLP group treated with saline (SAL). CLP-SAL group shows disarrangement of hepatocytes with diffuse microvacuolization by fat degeneration (arrows) and numerous apoptotic cells. Note that in CLP group treated with Gln, the histoarchitecture of the hepatocytes is restored with decreased apoptotic cells (arrowheads). **(Small intestine villi) **C group depicted preserved architecture with normal crypts (Cry) and villi (Vil) with few apoptotic cells. CLP presents necrosis of the top of villi (Nec), degenerative cytoplasmic changes of enterocytes (arrows), and numerous apoptotic cells. In CLP-Gln group, the histoarchitecture of the crypts and villi is restored with decrease of the apoptotic cells (arrowheads).

Small intestine villi, kidney, lung, and liver epithelial cell apoptosis were higher in CLP-SAL compared with C-SAL (Figures 4 and 6, and Table 3), while Gln attenuated epithelial cell apoptosis in kidney and lung, and avoided these changes in small intestine villi and liver. In CLP-SAL we observed glomerular lesion degeneration and vacuolization in the liver, and small intestine villi epithelial injury (Figure 6).

**Table 3:** Epithelial cell apoptosis

Groups	Lung	Kidney	Liver	Villi
C-SAL	0.0 (0 to 1)	0.0 (0 to 1)	0.0 (0 to 1)	0.0 (0 to 1)
C-Gln	0.0 (0 to 1)	0.5 (0 to 1)	0.5 (0 to 1)	0.0 (0 to 1)
CLP-SAL	2.5 (2 to 4)*	2.0 (2 to 3)*	2.0 (2 to 3)*	3.5 (3 to 4)*
CLP-Gln	1.5 (1 to 2)* #	1.0 (1 to 1)* #	0.5 (0 to 1)	0.0 (0 to 1)

Values are median (25^th ^to 75^th ^percentile) of five animals in each group. A five-point semiquantitative severity-based scoring system was used. The apoptotic findings were graded as: 0 = normal lung parenchyma; 1 = 1 to 25%; 2 = 26 to 50%; 3 = 51 to 75%; 4 = 76 to 100% of examined tissue. Sepsis was induced by cecal ligation and puncture surgery (CLP). A sham-operated group was used as control group (C) for animals undergoing CLP. One hour after surgery, C and CLP groups were further randomized into subgroups receiving saline (SAL) or glutamine (Gln). *Significantly different from C group (*P *< 0.05). #Significantly different from CLP-SAL group (*P *< 0.05).

**Figure 7:**
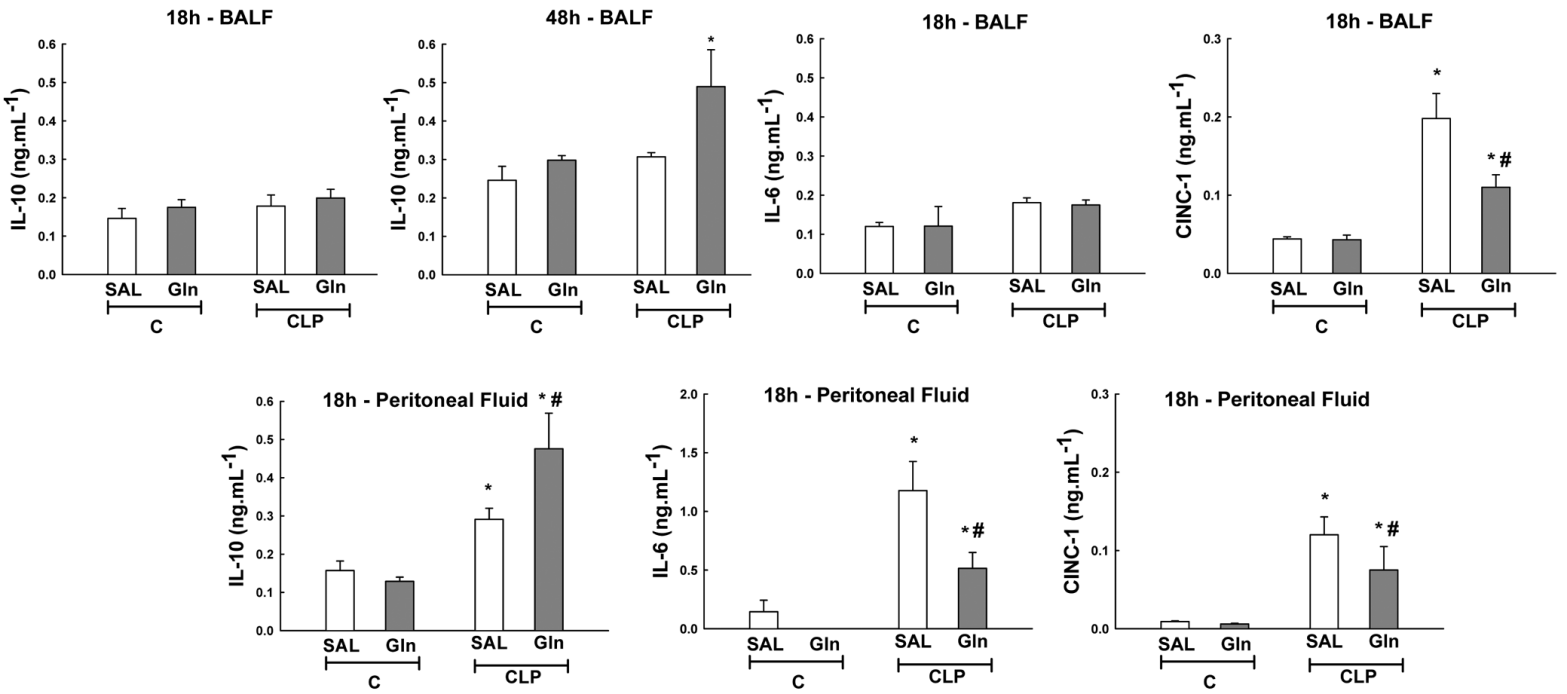
Analysis of CINC-1 (cytokine-induced neutrophil chemoattractant-1), IL-10 and IL-6 levels measured in both bronchoalveolar and peritoneal lavage fluids 18 and 48 hours after sepsis induction.  Values are ± standard deviation of five animals in each group. Sepsis was induced by cecal ligation and puncture surgery (CLP). A sham-operated group was used as control (C) for animals undergoing CLP. One hour after surgery, C and CLP groups were further randomized into subgroups receiving saline (SAL) or glutamine (Gln). *Significantly different from C group (*P *< 0.05). #Significantly different from CLP-SAL group (*P *< 0.05). BALF = bronchoalveolar lavage fluid.

Eighteen hours after surgery, CINC-1 levels increased in CLP-SAL compared to C-SAL in the broncho-alveolar lavage fluid and peritoneal lavage fluid, while Gln minimized these changes (Figure 7). However, no significant changes in CINC-1 were observed at 48 hours both in broncho-alveolar lavage fluid and peritoneal lavage fluid. At 18 hours, IL-10 and IL-6 were higher in CLP-SAL than C-SAL in the peritoneal lavage fluid, but similar in all groups in the broncho-alveolar lavage fluid. Gln reduced IL-6 in the peritoneal lavage fluid. At 48 hours, IL-10 increased in the CLP-Gln group in BALF and at 18 hours in the PLF (Figure 7). However, no significant changes were observed in IL-10 in the PLF at 48 hours.

## Discussion

In the present experimental model of polymicrobial sepsis induced by cecal ligation and puncture surgery in rats, one single early iv dose of Gln (0.75 g/kg) improved survival and oxygenation, prevented lung mechanics deterioration, and minimized pulmonary and diaphragm histological changes, attenuating epithelial cell apoptosis of the lung and distal organs. In addition, Gln acted on balancing pro- and anti-inflammatory cytokines, decreasing CINC-1 and IL-6 in BALF and PLF at 18 hours, and increasing IL-10 in PLF at 18 hours and BALF at 48 hours.

We used a CLP model of sepsis for the following reasons: it is reproducible and more comparable with human surgical sepsis; apoptosis of selected cell types and host immune responses seem to mimic the course of human sepsis [28]; and it is considered a good model for abdominal sepsis therapy research [28-30].

In our study, a single 0.75 g/kg dose of iv Gln was used as it resulted in a plasma Gln level of 3 to 7 mM/L in a model of endotoxemia [4]. This dose of Gln was found to markedly enhance HSP expression in lung attenuate proinflammatory cytokine release [4,11], and improve survival after endotoxemia [4,12,17].

In the present study, Gln led to a reduction in neutrophil infiltration, interstitial oedema, and alveolar collapse (Table 1), as well as a repair in alveolar capillary membrane (Figure 2 and Table 2) yielding an improvement in oxygenation, lung Est, ΔP1, and ΔP2 (Figure 1). The beneficial effects of iv Gln on pulmonary inflammation in experimental models of sepsis have been previously reported [11-13,17], but not directly related to gas-exchange and lung mechanics. Furthermore, no prior study has analysed the impact of Gln on the repair of the alveolar capillary membrane through electron or confocal microscopy. Therefore, the beneficial effects of Gln on lung parenchymal structure result in the improvement in clinical parameters (lung mechanics and gas exchange) which may lead to a less injurious setting of mechanical ventilation.

We also observed that Gln reduced *in vivo *epithelial cell apoptosis in lung, small intestine villi, kidney, and liver (Table 3). Emerging *in vitro *evidence showed that Gln deprivation may influence cell survival and gene expression [15,31-33]. Additionally, the effects of Gln on epithelial cell apoptosis have been studied mainly in intestinal [32-34] but not in lung cells. A recent *in vitro *study demonstrated that in intestinal cells, the role of extracellular signal-regulated kinase pathway in Gln-mediated prevention of cellular apoptosis following stress or injury [33]. The phosphoinositide-3 kinase/Akt pathway appears to be activated during periods of Gln starvation, which may serve as a protective mechanism to limit apoptosis associated with cell stress [34]. Additionally, other factors have been variably implicated in Gln-dependent survival signalling [15]. To date, no other studies have shown *in vivo *distal organ apoptosis after iv Gln therapy in sepsis.

Pro-inflammatory cytokines are primarily responsible for initiating an effect against exogenous pathogens. However, excessive production of these mediators may significantly contribute to shock and multiple organ failure [21]. In contrast, anti-inflammatory cytokines are crucial for down regulating the incremented inflammatory process and maintaining homeostasis for the correct function of vital organs. Therefore, a balance between pro- and anti-inflammatory cytokines is important for appropriate immune response; although excessive inflammation or hyporesponsiveness could lead to complications. The protective effects of Gln against apoptosis in lung and peripheral organs may also be attributed to the association of reduced pro-inflammatory cytokines (CINC-1 and IL-6) with an increase in anti-inflammatory cytokine (IL-10) in BALF and PLF (Figure 7). It has been reported that CINC-1 plays an important role in the recruitment of neutrophils to the lung in lipopolysaccharide-induced ALI [35]. The migration of blood neutrophils into the lung partially depends on chemokines such as IL-8 (human), CINC-1 (rat), and macrophage inflammatory protein-2. On the other hand, the lack of endogenous IL-10, a prototypic anti-inflammatory cytokine, resulted in increased levels of TNF and enhanced mortality in mouse models of endotoxemia, whereas in models of bacterial infection, endogenous IL-10 impairs the bacterial clearance [36]. Therefore, our data suggest that Gln's protective effects on lung and distal organ injury can also be explained by a better anti-inflammatory response and immune regulation.

Different mechanisms have been investigated to explain the potential protective effects of Gln against inflammatory injury, such as: attenuation of excessive NF-κB activation reducing the release of TNF-α, IL-6, and IL-18 in sepsis [11]; up regulation of HSP70 and HSP72 [12-17] repairing denaturated/injured proteins or promoting their degradation following irreparable injury; and increment in tissue glutathione levels, improving the antioxidant status [37]. Although these parameters were not measured in the present study, it is likely that these mechanisms are involved in the reduction of the distal organ inflammatory process.

Gln also limited diaphragm ultrastructural changes. Doruk and colleagues showed that Gln reversed the reduction in glutathione levels in the diaphragm of rats submitted to cecal ligation and puncture surgery [38]. However, no previous study has demonstrated the histological changes of diaphragm in Gln-treated sepsis model.

The current study has some limitations which need to be addressed. First, a CLP experimental model of sepsis was used [21]. The CLP is certainly a good model of peritonitis, and we do not know if these results can be directly shifted to other experimental models of sepsis. Second, the amount of bacteria recovered from peritoneal and blood samples was not measured. Third, only one single iv dose of Gln (0.75 g/kg) was used [4], and consequently, we cannot exclude the possibility that multiple doses or continuous infusion could yield better histological results [11]. Fourth, Gln was intravenously used; thus we do not know the effects of the 0.75 g/kg Gln dose via enteral route. Enteral Gln has a protective effect against lipopolysaccharide-induced mucosal injury [39], as well as ameliorates bacterial translocation, endotoxemia, apoptosis, and improves the ileal and liver histology in the presence of obstructive jaundice [40]. However, recently, it has been described that Gln leads to interstitial inflammation and fibrosis in lipopolysaccharide-induced ALI [41]. Furthermore, enteral administration of Gln may be questionable in peritonitis and does not improve survival in intensive care unit patients [42]. Fifth, Gln was given early after injury, and therefore, the use of Gln in the late phase of sepsis is unknown. Sixth, plasma Gln levels were not analyzed, although prior studies have shown reduced levels of Gln in plasma and muscle during sepsis [5,7,43]. Finally, we measured IL-10, IL-6, and CINC-1 in the BALF and PLF. However, the effects on other cytokines and their amount in lung tissue have not been investigated. Even taking into account all these limitations the present data demonstrate the beneficial effects of Gln in abdominal sepsis on lung as well as on diaphragm and distal organs.

## Conclusions

In the present experimental model of sepsis induced by cecal ligation and puncture, a single early iv Gln improved survival and arterial oxygenation, prevented pulmonary mechanics deterioration and minimized histological changes, attenuating epithelial cell apoptosis of the lung and distal organs. These findings suggest that Gln may modulate the inflammatory process reducing the risk of lung and distal organ injury. Thus our experimental data suggest that a single early iv dose of Gln could be beneficial to patients submitted to surgery for peritonitis, but this hypothesis must be proved in further clinical studies.

## Key messages

• The early use of iv Gln attenuated the histological changes and the increase in epithelial cell apoptosis of the lung, kidney, liver, and small intestine villi induced by abdominal sepsis.

• Its early use also improved oxygenation, prevented lung mechanics deterioration, and minimized diaphragm ultrastructural modifications.

• These beneficial effects can be determined by a balance between pro- and anti-inflammatory cytokines both in BALF and PLF.

• Gln infusion may be beneficial to patients submitted to surgery for peritonitis, but this hypothesis must be further proved in clinical studies.

## Abbreviations

ΔP1: resistive pressure; ΔP2: viscoelastic/inhomogeneous pressure; ALI: acute lung injury; ANOVA: analysis of variance; ARDS: acute respiratory distress syndrome; BALF: bronchoalveolar lavage fluid; C: control; CINC-1: Cytokine-Induced Neutrophil Chemoattractant; CLP: cecal ligation and puncture; ELISA: enzyme-linked immunosorbent assay; Est: static elastance; FiO_2_: fraction of inspired oxygen; Gln: glutamine; H&E: haematoxylin & eosin; HSP: heat shock protein; IL: interleukin; ip: intraperitoneal; iv: intravenous; NF-κB: nuclear factor-κB; PaO_2_: partial pressure of arterialoxygen; PEEP: positive end-expiratory pressure; PLF: peritoneal lavage fluid; Pplat: plateau; Req: flow resistance; Req/V': resistive pressure; TTF1: Thyroid Transcription Factor 1; TUNEL: Terminal deoxynucleotidyl Transferase Biotin-dUTP Nick End Labelling; V_T_: tidal volume.

## Competing interests

The authors declare that they have no competing interests.

## Authors' contributions

GPO: Animal preparation, performance of experimental work, analysis of the mechanical and histological data, statistical analysis, writing of the manuscript. MBGO: Animal preparation, performance of experimental work, preliminary analysis of the data, helped to draft the manuscript. RSS: Animal preparation, performance of experimental work, analysis of the mechanical data, helped to draft the manuscript. LDL: Animal preparation, performance of experimental work, analysis of the mechanical and morphometrical data. CMD: Animal preparation, performance of experimental work, analysis of the mechanical and morphometrical data, helped to draft the manuscript. AMAS: Analysis of the histological data, helped to draft the manuscript. WRT: Analysis of the histological data, helped to draft the manuscript. VLC: Analysis of the histological data, helped to draft the manuscript. RNG: Analysis of the immunological data (ELISA), helped to draft the manuscript. PTB: Analysis of the immunological data (ELISA), helped to draft the manuscript. PP: Experimental design, writing of the manuscript, supervision and overview of entire project. PRMR: Experimental design, supervision of experimental work, statistical analysis, writing of the manuscript, supervision and overview of entire project. All authors revised the manuscript and approved the final version.
